# Empathy, Pain and Attention: Cues that Predict Pain Stimulation to the Partner and the Self Capture Visual Attention

**DOI:** 10.3389/fnhum.2017.00465

**Published:** 2017-09-20

**Authors:** Lingdan Wu, Ursula Kirmse, Tobias Flaisch, Ganna Boiandina, Anna Kenter, Harald T. Schupp

**Affiliations:** Department of Psychology, University of Konstanz Konstanz, Germany

**Keywords:** attention, emotion, empathy, EPN, LPP

## Abstract

Empathy motivates helping and cooperative behaviors and plays an important role in social interactions and personal communication. The present research examined the hypothesis that a state of empathy guides attention towards stimuli significant to others in a similar way as to stimuli relevant to the self. Sixteen couples in romantic partnerships were examined in a pain-related empathy paradigm including an anticipation phase and a stimulation phase. Abstract visual symbols (i.e., arrows and flashes) signaled the delivery of a Pain or Nopain stimulus to the partner or the self while dense sensor event-related potentials (ERPs) were simultaneously recorded from both persons. During the anticipation phase, stimuli predicting Pain compared to Nopain stimuli to the partner elicited a larger early posterior negativity (EPN) and late positive potential (LPP), which were similar in topography and latency to the EPN and LPP modulations elicited by stimuli signaling pain for the self. Noteworthy, using abstract cue symbols to cue Pain and Nopain stimuli suggests that these effects are not driven by perceptual features. The findings demonstrate that symbolic stimuli relevant for the partner capture attention, which implies a state of empathy to the pain of the partner. From a broader perspective, states of empathy appear to regulate attention processing according to the perceived needs and goals of the partner.

## Significance Statement

We report that symbolic stimuli predicting painful stimuli to the partner elicit the brain signature of attentive processing, as indicated by early posterior negativity (EPN), and late positive potential (LPP) components. This work suggests that states of empathy may facilitate attention processing according to perceived needs and goals of the partner.

## Introduction

The detection of significant stimuli leads to a characteristic ERP sequence. Numerous studies have shown that the processing of emotionally significant as compared to neutral stimuli is associated with a relative EPN between 150 ms and 350 ms and a LPP between 350 ms and 700 ms. These ERP components are presumed to reflect the preferential processing and attention capture of significant stimuli (Schupp et al., 2006; Bradley, 2009; Mühlberger et al., 2009). In the present study, these two ERP components sensitive to emotional significance were used to explore attention processes in the domain of empathy.

The concept of empathy refers to a multifaceted phenomenon with the core meaning “understanding and feeling with somebody” (Bischof-Köhler, 2012; Preston and Hofelich, 2012; Zaki, 2014; Cuff et al., 2016). It is a strong motivator for helping and cooperative behavior and accordingly serves an important function in the social life of humans. Here, empathy was examined with respect to its effects on attention processes. We deemed that in a state of empathy, stimuli significant to other people become the focus of attention and modulate ERP components sensitive to stimulus significance in similar ways as stimuli relevant to the self.

Previous research provides first evidence for this notion. Specifically, a number of studies have assessed the processing of empathy-related picture contents, e.g., a knife cutting a hand or a facial expression of strong pain, as compared with carefully selected control stimuli. Findings consistently demonstrate that the LPP component was larger for empathy-related picture contents (Fan and Han, 2008; Decety et al., 2010; Li and Han, 2010; Meng et al., 2013). However, from a theoretical perspective, what the brain signature of attention specifies remains ambiguous. Specifically, it is assumed that the LPP reflects the activation of an emotional memory representation in long-term memory by a corresponding stimulus input (Lang, 1993; Schupp et al., 2006, 2016; Bradley, 2009). Thus, it is possible that the LPP response to empathy-related pictures reflects the presence of emotionally diagnostic perceptual features in the images. In order to provide stronger evidence for the attention capture of other-relevant stimuli, the sensory-perceptual and implied meaning units of the emotion network need to be dissociated.

The pain-related empathy paradigm developed by Singer et al. (2004, 2006, 2008) allows studying the hypothesis that a state of empathy guides attention towards stimuli significant to others while controlling for perceptual features of the stimulus materials. Assuming that empathy is amplified towards a loved one, functional imaging data were collected while either the participant or their romantic partner received a Pain or Nopain electric stimulus, which was signaled by an abstract visual cue. The findings showed that brain regions activated when receiving the shock directly, i.e., anterior insula, dorsal anterior cingulate cortex, brain stem and cerebellum, were also activated in the empathy condition, i.e., when the partner received the shock. Furthermore, providing first evidence that empathy may guide attention processes, the findings showed that pain compared to non-painful stimuli were associated with increased activations in visual associative regions (Singer et al., 2004).

In studying pain and pain-related empathy processes, it is critical to distinguish between the anticipation of pain and the experience of pain. While the experience of pain facilitates escape behaviors minimizing immediate harm, expectation of pain can prevent future harm. In accordance with this notion, functional imaging studies revealed distinct neural activations for the anticipation of pain compared to pain experience in medial frontal regions, insula and cerebellum (Ploghaus et al., 1999). Meta-analysis suggests an even more extended set of brain regions, including the dorsolateral prefrontal, midcingulate and anterior insula cortices, medial and inferior frontal gyri, inferior parietal lobule middle and superior temporal gyrus, thalamus and caudate (Palermo et al., 2015). Furthermore, ERP studies showed that visual stimuli signaling the possibility for receiving an electric shock modulated P1, P2 and LPP components, thought to reflect sensitized perceptual processing and increased selective attention effects (e.g., Baas et al., 2002; Böcker et al., 2004; Bublatzky and Schupp, 2012). Distinct effects were observed for the actual delivery of painful stimuli, reflecting differences in stimulus modality and intensity. Specifically, pain stimulation elicits pronounced P150–N260 and P3 components, with the P150–N260 complex closely tracking the perceived intensity of pain stimuli (Chen et al., 1979; Miltner et al., 1988a,b). Overall, functional imaging and ERP studies indicate that anticipation and experience of pain for the self are associated with distinct effects, which need to be considered when studying attention processes in the context of pain-related empathy.

The main goal of the present study was to examine the hypothesis that empathy regulates attention processes. Towards this end, we relied on the pain-related empathy paradigm (Singer et al., 2004) in which couples in romantic partnerships were exposed to abstract visual stimuli (anticipation phase) predicting the delivery (stimulation phase) of a Pain or Nopain (Intensity) stimulus to the partner or the self (Target). For theoretical and methodological reasons, the anticipatory phase is of primary interest. Specifically, the information value of the anticipatory cue is higher compared to the stimulation cue indicating that the partner receives the stimulation. In addition, the self-other comparison is only meaningful for the anticipatory condition since the stimulation period includes different stimuli for the couple (visual cue vs. visual cue plus pain stimuli). According to the hypothesis that a state of empathy guides attention processes, we expected that anticipatory cues signaling Pain stimuli to the partner elicit larger EPN and LPP components than cues signaling Nopain stimuli. Furthermore, the ERP components indicating attentive processing mediated by empathic involvement were predicted to be similar in appearance to the EPN and LPP modulations elicited by cues predicting self-related Pain stimuli. In addition, based on the notion that self-relevant stimuli are particularly efficient in capturing attention (Conway et al., 2016), larger EPN and LPP components were predicted in the self-relevant condition. For the stimulation phase, enhanced N150–P260 and P3 components were expected during the delivery of the Pain as compared to the Nopain stimulus to the self. However, these effects were assumed to be specific to the Self-condition, since viewing others’ pain does typically not invoke the sensory components of pain processing according to fMRI studies (Singer et al., 2004, 2008).

## Materials and Methods

### Participants

Participants were 16 heterosexual couples between the ages of 18 years and 25 years (*M* = 21.0; SD = 1.9). The average duration of their relationship was 40.6 (SD = 26.8, ranging from 4 to 108) months. The average score of relationship quality based on the ratings of closeness of relationship questionnaire was 26.80 (SD = 4.24; Range: 16–32). A further couple was not analyzed because debriefing indicated that one participant misunderstood the instructions. All participants were healthy at the time of measurement, reported no history of neurological or psychiatric disorders, and had normal or corrected to normal vision. They received monetary compensation or course credit for participation. This study was carried out in accordance with the recommendations of ethical guidelines by the ethical committee of the University of Konstanz. All participants gave written informed consent in accordance with the Declaration of Helsinki. The protocol was approved by the ethical committee of the University of Konstanz.

### Stimulus Materials

#### Visual Stimuli

**Figure 1:**
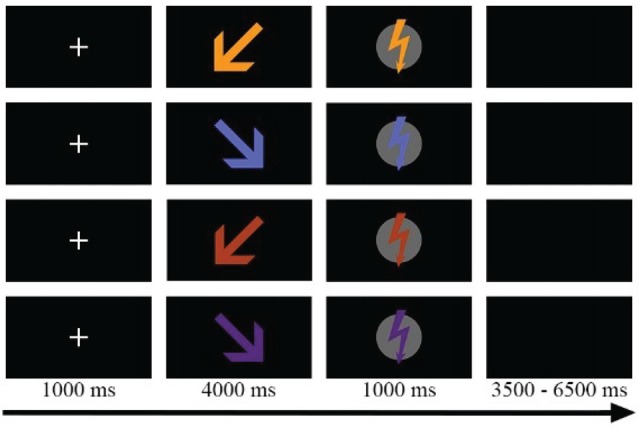
Sample trials of the four experimental conditons, e.g., Self-Pain, Partner-Pain, Self-Nopain, Partner-Nopain. Target of the stimulation was indicated by the direction of the arrow (left vs. right). Color of the arrow indicated the intensity of the stimulation (Pain vs. Nopain). In the stimulation phase, a flash of the same color as the preceding arrow was shown to indicate electric stimulus delivery. Assignment of colors was balanced for target and intensity across participants.

As shown in Figure 1, eight different stimuli were presented in two phases: an anticipation phase and a stimulation phase. During the anticipation phase, an arrow (430 × 430 pixels) was presented to announce the target (Self vs. Partner) and the painfulness (Pain vs. Nopain) of the upcoming electric stimulation. The direction of the arrow indicated the target of stimulation. Inclined at an angel of 45°, arrows pointing towards the left or right side signaled which person (left vs. right) would be the target of the upcoming Pain or Nopain stimulus in this trial. The color of the arrow indicated the intensity of the stimulation (Pain vs. Nopain) with different colors presented to the participants to prevent conditioned responses, i.e., red and orange presented to one participant and blue and purple to the other. During the subsequent stimulation phase, a vertically oriented flash (400 × 430 pixels) of the same color as the preceding arrow was presented to indicate the actual administration of the electric stimulus. In order to control for differences in the physical stimulus characteristics of the visual stimuli, the assignment of the colors of the visual symbols was balanced for target and intensity across participants. The visual stimuli were presented via Presentation software (Neurobehavioral Systems, Inc., Albany, CA, USA) onto a 21-inch cathode ray tube (CRT)-monitor at a viewing distance of 160 cm.

#### Electric Stimulation

Electric stimuli consisted of a low (“Nopain”) and a high (“Pain”) intensity stimulus which were generated by a custom-built stimulator and transmitted via a constant current unit (CCU1) to an Ag/AgCl electrode attached to the back of the hand. A 1000-ms train of impulses was generated by a square-wave generator (square pulse of 5 ms length and 100 Hz frequency). The high intensity stimulus (*M* = 3.10 mA; SD = 1.37) was individually adjusted to a level that was experienced as unpleasant and painful. To this end, each participant was asked to rate a series of electric stimulations that began with imperceptible intensity (0.5 mA) and increased in 0.3 mA steps until the individually tolerable level was reached.

### Procedure

After arrival at the laboratory, the couple was informed about the study and asked to read and sign the informed consent. Then, in separate rooms, participants individually adjusted the intensity of the electric stimuli. Afterwards, participants were seated in the experimental room next to each other and dense sensor EEG nets were attached to both of them. A screen prevented the participants from seeing each other to control for effects associated with looking at the partner (Master et al., 2009; Eisenberger et al., 2011). Afterwards, participants were instructed that during the experiment, a series of abstract visual symbols (i.e., arrows and flashes) and electric pulses would be presented, with the arrows indicating the target and intensity of the upcoming electric stimulation and the flashes indicating the target and intensity of the currently administered electric stimulation. Participants were asked to refrain from talking during the experiment. Prior to the main experiment, four practice trials (one per condition) were given to familiarize the participants with the experimental stimuli and the procedure. The whole experiment consisted of two blocks with 20 trials for each of the four conditions (i.e., Self-Pain, Self-Nopain, Partner-Pain and Partner-Nopain) delivered in a random order, resulting in a total of 160 trials. Each trial began with the presentation of a central fixation cross (1 s), followed by the presentation of an arrow stimulus (4 s) in the anticipation phase, announcing who would receive the electric stimulation and whether it would be painful or not painful. In the subsequent stimulation phase, a same colored flash was presented, and the electric stimulation was released for 1 s. The inter-trial interval was 3.5–6 s. The whole experiment lasted for about 30 min. To validate the experimental procedure, self-report measures of experienced pain during electric stimulation were obtained after each block. Specifically, the pain experienced during reception of the low and high intensity stimuli was evaluated on a scale from 1 (not painful) to 7 (very painful). Furthermore, participants were asked to rate how unpleasant they felt when the electric stimulations were given to either themselves or their partners (1 = not unpleasant, 7 = very unpleasant). Following the main experiment, participants completed the German version of Interpersonal Reactivity Index, which provides an assessment of empathy (SPF; Paulus, 2009)^1^. Furthermore, the Couple chemistry rating (PKS, Schneewind and Kruse, 2002), and the Inclusion of Other in the Self Scale (IOS; Aron et al., 1992) were used to assess closeness of relationship.

### ERP Data Acquisition and Analysis

Brain and ocular scalp potential fields were measured with a 129-channel geodesic sensor net (EGI: Electrical Geodesics Inc., Eugene, OR, USA), on-line bandpass filtered from 0.01 Hz to 100 Hz and sampled at 250 Hz using EGI Geodesic amplifiers and Netstation acquisition software. Electrode impedance was kept below 40 kΩ, as recommended for this type of electroencephalogram (EEG) amplifier by EGI guidelines. Data were recorded continuously with the vertex sensor as reference electrode. A 40 Hz digital low pass filter was applied off-line to the continuous EEG data. The reported data were corrected for ocular artifacts based on a multiple regression method (Miller et al., 1988), converted to an average reference, and baseline-adjusted (100 ms pre-stimulus). Data editing and artifact rejection were performed based on an elaborate method for the statistical control of artifacts, specifically tailored for the analyses of dense sensor ERP recordings (Junghöfer et al., 2000). Finally, separate average waveforms were calculated for the anticipation and stimulation phases according to the four experimental conditions. Applying strict artifact criteria, on average 18.3% (SD = 3.5%) of the trials were excluded from calculating the average waveforms, which did not differ across experimental conditions (*F*_(3, 93)_ = 1.63, *p* = 0.19, *η*^2^ = 0.05).

### Anticipation Phase

Visual inspection served to identify effects due to *Target* (Self vs. Partner) and *Stimulus Intensity* (Pain vs. Nopain). Accordingly, EPN and LPP components were computed as mean activity in selected sensor regions and time intervals.

To capture the EPN effects, the mean activity over a time interval of 260–320 ms was calculated in a temporo-occipital sensor cluster (EGI sensor numbers: 57, 58, 59, 63, 64, 65, 66, 69, 70, 71, 72, 74, 75, 77, 83, 84, 85, 89, 90, 91, 92, 95, 96, 97, 100, 101).

As illustrated in Figure 4, the temporal development of the LPP effects had a somewhat different appearance for Self- and Partner-conditions. Accordingly, the amplitude of the LPP was scored as mean activity over two time intervals, i.e., 400–600 ms and 600–800 ms, in a centro-parietal sensor cluster (EGI sensor numbers: 7, 31, 32, 38, 53, 54, 55, 60, 61, 62, 67, 68, 78, 79, 80, 81, 86, 87, 88, 106, 107, 129). In addition to the modulation of the amplitude of the LPP component, the effect of Target also showed a modulation at the onset of the LPP. Accordingly, a further analysis was conducted capturing the onset of the LPP in a time interval from 350 ms to 400 ms.

**Figure 2:**
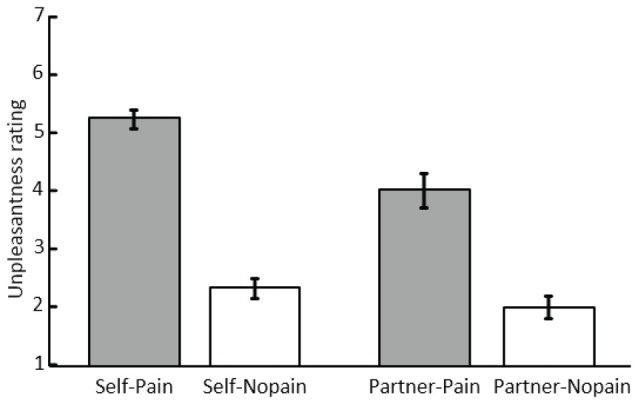
Mean scores and standard error of unpleasantness ratings under the “Self-Pain”, “Self-Nopain”, “Partner-Pain” and “Partner-Nopain” conditions.

**Figure 3:**
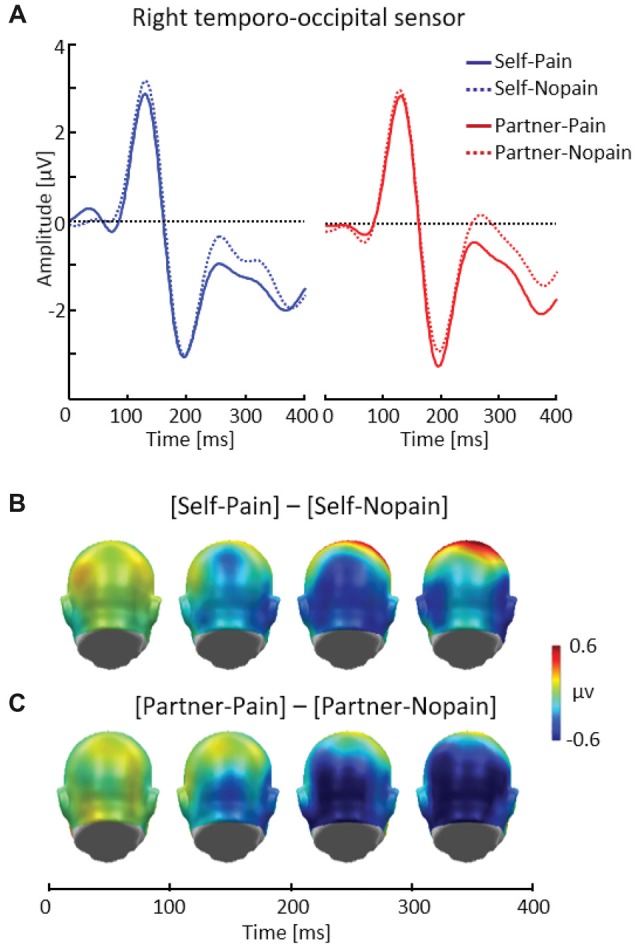
Illustration of the early posterior negativity (EPN) component showing a representative right temporo-occipital sensor (EGI # 90) **(A)**. Scalp potential maps of the difference waves of [Self-Pain] — [Self-Nopain] **(B)**, [Partner-Pain] — [Partner-Nopain] conditions **(C)**. A back view of the the model head is shown.

**Figure 4:**
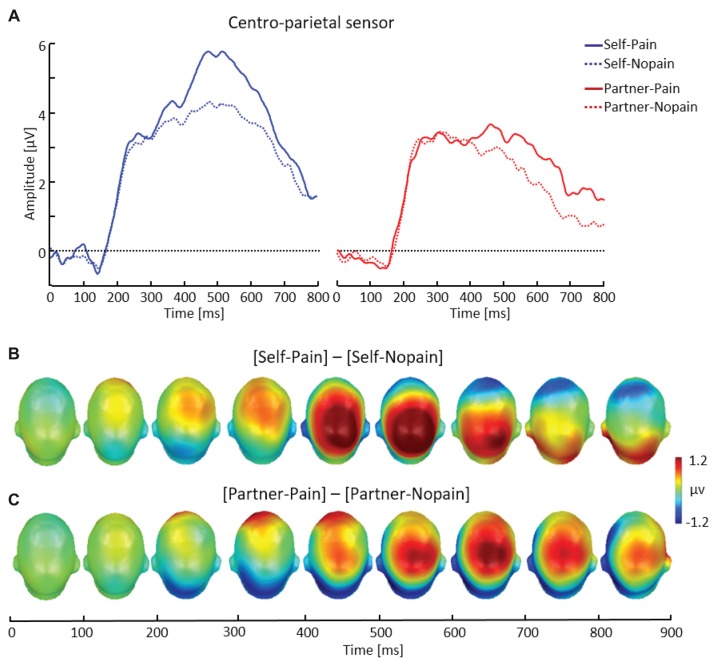
Illustration of the late positive potential (LPP) component showing a representative centro-parietal sensor (EGI # 54) **(A)**. Scalp potential maps of the difference waves of [Self-Pain] — [Self-Nopain] **(B)**, [Partner-Pain] — [Partner-Nopain] conditions **(C)**. A top view of the model head is shown.

Both ERP components were submitted separately to repeated-measures ANOVAs, including the within subject factors Target (Self vs. Partner) and Stimulus Intensity (Pain vs. Nopain). The LPP analysis included the additional factor Time Interval (400–600 ms vs. 600–800 ms). Significant main effects and interactions were followed up with paired-samples *t*-tests.

### Stimulation Phase

While the visual stimuli were the same for Self- and Partner-conditions, the Self-condition included the delivery of the electric stimuli. To acknowledge these differences in experimental procedure, Self- and Partner-conditions were examined separately. Consistent with previous research (e.g., Miltner et al., 1988a), pronounced effects of *Stimulus Intensity* were observed in the Self-condition for the N150, P260 and P3 components.

#### Self-Condition

To capture the N150 effect, the mean activity over a time interval of 145–175 ms was calculated in fronto-central sensor clusters (EGI sensor numbers: 6, 7, 107, 129). The P260 amplitude was also scored in the same fronto-central clusters and was most pronounced in a time interval from 215 ms to 245 ms. Previous research has shown that the N150–P260 difference covaries with the intensity of Pain stimuli (Chen et al., 1979; Miltner et al., 1988a,b). Accordingly, difference scores between the peak amplitudes of the N150 over the time interval of 145–175 ms and the P260 over the time interval of 215–245 were calculated.

To assess the P3 effect, the mean activity over a time interval of 280–400 ms was calculated in a centro-parietal sensor cluster (EGI sensor numbers: 7, 31, 32, 38, 53, 54, 55, 60, 61, 62, 67, 68, 78, 79, 80, 81, 86, 87, 88, 106, 107, 129).

#### Partner-Condition

In contrast to the Self-condition, visual inspection did not suggest differences in the processing of the visual cue that indicated the actual delivery of Pain or Nopain stimuli to the partner. As there were no apparent effects in the Partner-condition, the main analysis focused on the Self-condition by submitting the N150–P260 and P3 components of Pain vs. Nopain stimuli to separate *t*-tests.

For effects involving repeated measures, the Greenhouse–Geisser procedure was used to correct for violations of sphericity.

## Results

### Self-Report

#### Unpleasantness Ratings

As shown in Figure 2, in the Self-condition, Pain stimuli (*M* = 5.23; SD = 0.89) were rated as more unpleasant than Nopain stimuli (*M* = 2.31; SD = 0.93). Interestingly, a similar, albeit less pronounced effect was observed when the partner was the target of the stimulation. Specifically, in the Partner-condition, Pain stimuli (*M* = 4.00; SD = 1.64) were rated as more unpleasant than Nopain stimuli (*M* = 1.98; SD = 1.11). Statistical analysis supported these observations by revealing significant main effects of *Target* and *Stimulus Intensity*, *F*_(1, 31)_ = 12.86 and 140.30; *p*s < 0.01, *η*^2^ = 0.29 and 0.82, as well as a significant interaction of both factors, *F*_(1, 31)_ = 18.85; *p* < 0.01, *η*^2^ = 0.38. Follow-up tests indicated that the Pain-condition was perceived as more unpleasant than the Nopain-condition in both the Self-condition, *t*_(31)_ = 13.79, *p* < 0.01, and the Partner-condition, *t*_(31)_ = 7.98, *p* < 0.01. Furthermore, the Pain stimuli were more unpleasant in the Self-condition compared to the Partner-condition, *t*_(31)_ = 4.60, *p* < 0.01. In contrast, the Nopain-conditions were rated similarly low in unpleasantness in the Self- and Partner-conditions, respectively, *t*_(31)_ = 1.55, *p* = 0.13.

#### Pain ratings

As expected, experienced pain during electric stimulation was significantly larger for the Pain (*M* = 4.13, SD = 1.04) than the Nopain-condition (*M* = 1.55, SD = 0.61), *t*_(31)_ = 11.97, *p* < 0.01.

### Event-Related Potentials

#### Anticipation Phase

##### EPN

Figure 3 shows that being the target of Pain stimuli was associated with a relative negative potential shift over posterior sensor regions compared to the Nopain stimuli. Interestingly, a similar pattern of EPN modulation with regard to time, topography and latency was observed in the Partner-condition. Specifically, the arrow cue announcing an upcoming painful stimulus to the partner also elicited a negative shift over posterior regions compared to the Nopain stimuli. Furthermore, comparing the ERP waveforms in the right and left panel of Figure 3A shows that the Self-condition elicited a relatively more negative potential than the Partner-condition, irrespective of stimulus intensity.

Repeated measure ANOVA revealed significant main effects of *Target*, *F*_(1, 31)_ = 6.41, *p* < 0.05, *η*^2^ = 0.17, and *Stimulus Intensity*, *F*_(1, 31)_ = 14.37, *p* < 0.01, *η*^2^ = 0.32, while the interaction of both factors was not significant, *F*_(1, 31)_ = 0.41, *p* = 0.53, *η*^2^ = 0.01. Exploratory *t*-tests confirmed the effect of *Stimulus Intensity* for both the Self (Pain: *M* = −0.96, SD = 1.62, Nopain: *M* = −0.47, SD = 1.70) and the Partner-condition (Pain: *M* = −0.58, SD = 1.61, Nopain: *M* = 0.10, SD = 1.51), *t*_(31)_ = −2.06 and −3.43, respectively, *p*s < 0.05.

##### LPP

As shown in Figure 4, arrow cues announcing a Pain stimulus for the self elicited a larger LPP over centro-parietal sensor regions compared to Nopain stimuli. Interestingly, a qualitatively similar effect was also seen in the Partner-condition, i.e., when the arrow cues indicated the intensity of the upcoming stimulus for the partner. However, the effect appeared to be accentuated in amplitude and somewhat earlier in time for the Self- as compared to the Partner condition.

##### LPP amplitude

Repeated measure ANOVA revealed a significant three-way interaction of *Target* × *Stimulus Intensity* × *Time Interval*, *F*_(1, 31)_ = 11.03, *p* < 0.01, *η*^2^ = 0.26 Accordingly, separate analyses were conducted for the two time intervals. For the earlier time interval (400–600 ms), main effects for Target, *F*_(1, 31)_ = 43.40, *p* < 0.01, *η*^2^ = 0.58, and *Stimulus Intensity*, *F*_(1, 31)_ = 23.03, *p* < 0.01, *η*^2^ = 0.47) were qualified by a significant interaction of both factors, *F*_(1, 31)_ = 6.87, *p* < 0.05, *η*^2^ = 0.18. Follow-up tests indicated larger LPP amplitudes elicited by Pain compared to Nopain cues for the Self-condition (Pain: *M* = 4.47, SD = 2.35, Nopain: *M* = 3.28, SD = 1.44), *t*_(31)_ = 4.65, *p* < 0.01, as well as the Partner-condition (Pain: *M* = 2.73, SD = 1.54, Nopain: *M* = 2.24, SD = 1.51), *t*_(31)_ = 2.78, *p* < 0.01. Furthermore, the difference in LPP amplitudes to Pain vs. Nopain stimuli was larger in the Self- compared to the Partner-condition, *t*_(31)_ = 2.62, *p* < 0.05. Analysis of the later LPP time interval (600–800 ms) revealed main effects for Target, *F*_(1, 31)_ = 17.72, *p* < 0.01, *η*^2^ = 0.36, and *Stimulus Intensity*, *F*_(1, 31)_ = 11.98, *p* < 0.01, *η*^2^ = 0.28). However, the interaction of the two factors was not significant, *F*_(1, 31)_ = 0.67, *p* = 0.42, *η*^2^ = 0.02. Follow-up tests indicated larger LPP amplitudes elicited by Pain compared to Nopain cues for the Self-condition (Pain: *M* = 2.40, SD = 1.45, Nopain: *M* = 1.93, SD = 1.09), *t*_(31)_ = 2.05, *p* < 0.05, as well as the Partner-condition (Pain: *M* = 1.59, SD = 1.40, Nopain: *M* = 0.89, SD = 1.28), *t*_(31)_ = 3.32, *p* < 0.01.

##### LPP onset (350–400 ms)

Analysis of the onset of the LPP component revealed a significant main effect of *Target*, *F*_(1, 31)_ = 13.60, *p* < 0.01, *η*^2^ = 0.31, indicating larger LPPs in the Self- compared to the Partner-condition. Furthermore, the interaction of *Target* by *Stimulus Intensity* approached significance, *F*_(1, 31)_ = 3.20, *p* = 0.08, *η*^2^ = 0.09. Follow-up *t*-tests indicated that the LPP onset was larger for Pain (*M* = 3.64, SD = 2.43) than for Nopain cues (*M* = 3.12, SD = 1.83) in the Self-condition, (*t*_(31)_ = 2.01, *p* = 0.05), while there was no significant difference between Pain (*M* = 2.68, SD = 1.91) and Nopain stimuli (*M* = 2.76, SD = 2.08) for the Partner-condition (*t*_(31)_ = −0.35, *p* = 0.73).

#### Stimulation Phase

##### N150–P260

**Figure 5:**
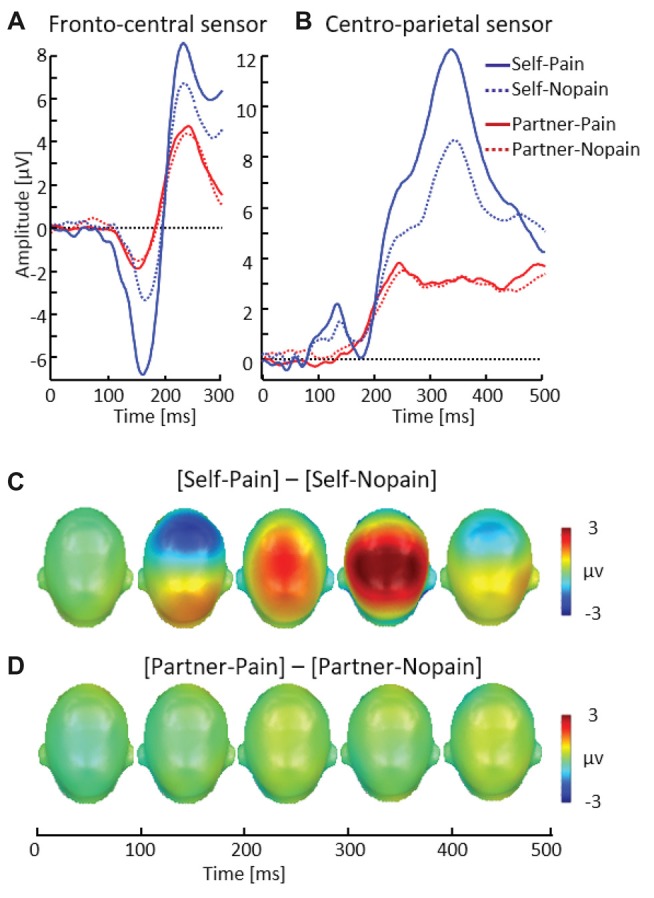
Illustration of the N150 and P260 components showing a representative fronto-central sensor (EGI # 7) **(A)**. Illustration of the P3 component showing a representative centro-parietal sensor (EGI # 54) **(B)**. Scalp potential maps of the difference waves of [Self-Pain] — [Self-Nopain] **(C)**, [Partner-Pain] — [Partner-Nopain] conditions **(D)**. A top view of the model head is shown.

In the Self-condition, participants simultaneously viewed a flash stimulus indicating the intensity and target of the stimulation and received tactile stimulation. Consistent with previous research examining processing of pain stimuli, the Pain (multi-compound) stimuli elicited a larger N150–P260 (*M* = 13.79, SD = 7.23) than the Nopain stimuli (*M* = 8.74, SD = 5.61; see Figure 5), *t*_(31)_ = 5.82, *p* < 0.01. This finding contrasts with the Partner-condition, in which participants received no tactile stimulation.

##### P3

As shown in Figure 5, the P3 revealed a similar pattern of modulation as observed for the N150–P260 peak. Specifically, in the Self-condition, the P3 was enhanced to the Pain compared (*M* = 8.43, SD = 3.67) to Nopain (multi-compound) stimuli (*M* = 5.97, SD = 3.31), *t*_(31)_ = 7.87, *p* < 0.01^2^.

## Discussion

The findings from the present study support the notion that a state of empathy facilitates attention towards stimuli significant for the partner. Specifically, during the anticipation phase, the processing of symbolic cues predicting the delivery of a Pain stimulus to the partner was associated with an EPN and LPP compared to cues indicating a Nopain stimulus. Notably, these effects are similar to the attention capture by arbitrary cues which predicted Pain stimuli for the self. The dissociation between anticipation and stimulation phases indicates that the meaning of the stimulus situation is critical for the observed findings. Specifically, in contrast to the anticipation phase, there was no ERP difference in processing the stimuli indicating the actual delivery of a Pain or Nopain stimulation to the partner. Overall, these findings show that significant stimuli to the partner become the focus of attention, as indicated by the EPN and LPP, components that were also observed in the processing of self-relevant stimuli.

With regard to cues signaling self-relevant conditions, the paradigm incorporates key elements of classical conditioning and social learning paradigms (Olsson and Phelps, 2004). Because participants are informed about the meaning of the cue stimuli, the procedure resembles social learning paradigms using verbal instructions to prime defensive response programs for upcoming aversive events and danger cues. In addition, as in classical conditioning, the participants have direct experience of the cue and the aversive pain stimulus. A wealth of research indicates that fear learning is associated with potentiated startle reflexes, enhanced electrodermal activity, heart rate responses and increased BOLD responses in core emotion regions, i.e., amygdala, insula, and anterior cingulate cortex in both classical conditioning (Hodes et al., 1985; Hamm and Weike, 2005; Weike et al., 2007; Sehlmeyer et al., 2009) and social learning paradigms (Grillon et al., 1991; Grillon and Davis, 1995; Funayama et al., 2001; Phelps et al., 2001; Olsson and Phelps, 2004; Bradley et al., 2005; Dalton et al., 2005; Mechias et al., 2010; Costa et al., 2015). Furthermore, event-related potential (ERP) studies demonstrated that conditioned stimuli and threat-cues modulate ERP components in early and later processing (Baas et al., 2002; Bublatzky and Schupp, 2012; Miskovic and Keil, 2012; Steinberg et al., 2012; Junghöfer et al., 2017). The present results replicate and extend these findings by demonstrating that arbitrary cues which announce a pain stimulus and are devoid of any a-priori emotional meaning do modulate ERP components typically associated with the processing of emotionally significant stimuli, i.e., EPN and LPP components. On the one hand, it conclusively demonstrates that these indices of stimulus significance are not necessarily dependent on specific physical stimulus features. On the other hand, establishing the signature of attentive processing for self-relevant stimuli serves as validation of the experimental paradigm and, within one experimental context, provides a template of comparison for empathy-related attention effects.

If enhanced attentive processing only occurred with respect to self-related stimuli, there would be no need to differentiate between the two colors signaling an upcoming Pain or Nopain stimulus for the partner. However, if empathic involvement also determines what is being attended, arbitrary visual cues can become significant when they reveal important information concerning the partner. The findings support the latter view. As foundation, the self-report data indicate empathic involvement for the partner showing significantly higher unpleasantness ratings when the partner receives Pain compared to Nopain stimuli. Most important, presumed to reflect increased visual attention, the EPN and LPP components are significantly larger to a cue signaling that the partner will receive Pain rather than Nopain stimuli. Notably, albeit attenuated (early LPP time interval) and, in the case of the LPP somewhat delayed (~50 ms), the EPN and LPP modulation of Pain stimuli appeared with similar latency and topography in the Partner- as observed in the Self-condition. Accordingly, the data are consistent with the notion that a state of empathy mediates the attention capture of a symbolic cue signaling the delivery of a Pain stimulus to the partner. Verbal instruction about the cue-stimulation assignment provides the grounds for understanding the affective meaning for and feeling with the partner. Within the given social context, stimuli relevant for the partner become meaningful and draw attention in similar ways as self-relevant stimuli. From a broader perspective, states of empathy appear to regulate attention processes according to the perceived needs and goals of the partner.

The findings revealed a dissociation for partner-related cues between the anticipation and stimulation phases. Contrary to the anticipation phase, an arrow signaling the actual delivery of a Pain or Nopain stimulus was not processed differentially. This finding is presumably specific to the experimental context in that the flash stimulus was coincident with the electric stimulation. One hypothesis is accordingly that the brain only selects arbitrary cues for enhanced processing if those stimuli are informative for subsequent adaptive behavior suited to remedy potential consequences. Future studies on the regulation of attention by empathy may examine this hypothesis by introducing the possibility for controlling adverse events delivered to the partner. Incidentally, the dissociation of findings between the anticipation and stimulation phases rules out an alternative hypothesis attributing the current findings to unspecific responses to any differences in stimulus intensity. The arrow and the flash signaled the identical difference in stimulus intensity, yet, the ERP modulation was specific to the anticipation phase. Thus, this finding is difficult to reconcile with an unspecific intensity hypothesis. Rather, it supports the notion that stimulus meaning and feeling for the partner are reflected in the ERP modulation during the anticipation phase.

The present study extends previous research on neural correlates of empathic processing reliant on pictures depicting pain-related contents. A consistent finding across studies was that pain-related pictures elicit a larger LPP compared to no-pain control stimuli (Fan and Han, 2008; Decety et al., 2010; Li and Han, 2010; Meng et al., 2013). For interpreting these effects, it was assumed that the observed effects are due to the empathic processing of somebody else’s pain. However, it was unclear whether this finding is specifically attributable to attentive processing mediated by empathy or reflects the sensory-perceptual features of the pictures diagnostic of emotional significance (see also Schupp et al., 2016). The present study disambiguates these two alternatives and provides strong evidence for the guidance of attention by a state of empathy using symbolic stimuli free of emotion-related sensory-perceptual features. Making arbitrary cues significant for the partner (and the self) by verbal instruction about the social condition lead to enhanced EPN and LPP components, similar to previous research relying on natural scenes depicting contents related to emotional experience in humans.

Functional imaging studies consistently revealed that perceiving pain in others activates regions in the anterior insula and anterior cingulate cortex, which are also activated when experiencing painful stimuli (Singer et al., 2004, 2006). There is some debate about the meaning of the overlap in these neural regions (Iannetti et al., 2013; Zaki et al., 2016). It is interesting, however, that these regions are key structures of the saliency network (Seeley et al., 2007), which is implicated in the regulation of attention and working memory across large-scale neural networks (Medford and Critchley, 2010; Menon and Uddin, 2010). The emerging view is, accordingly, that neither these brain regions, i.e., anterior insula and anterior cingulate cortex, nor the ERP components of attentive processing are specific markers for empathy (Zaki and Ochsner, 2012). Corroborating this notion, modulations of the EPN and LPP have been observed in several socially relevant contexts including for instance receiving feedback from a human sender compared to intelligent computer system (Schindler and Kissler, 2016) and the viewing of pictures depicting “loved” persons compared to control images (Guerra et al., 2011). Thus, a state of empathy may encompass neural regions devoted to detect and respond to significant stimuli in the environment with the present findings demonstrating that even arbitrary cues become efficient attention catchers when they are relevant for the partner.

EPN and LPP effects to Pain vs. Nopain stimuli were larger for the Self- compared to the Partner-condition. These findings correspond to previous ERP research demonstrating a processing advantage of self-relevant stimuli (Gray et al., 2004; Herbert et al., 2011; Bublatzky et al., 2014; Wieser et al., 2014; Fields and Kuperberg, 2015). Interestingly, there was a difference across processing time, with the EPN component showing a simple main effect of stimulus intensity with larger EPN amplitudes to Pain than to Nopain stimuli, while the LPP component revealed an interaction of target by stimulus intensity reflecting the largest LPP amplitudes to cues predicting Pain stimuli for the self. These findings corroborate previous research (Bublatzky and Schupp, 2012) indicating that with increasing stimulus processing time, effects of self-relevance become increasingly specific to threat stimuli.

In summary, the enhanced attention to abstract symbolic cues that predict upcoming pain of the partner implies that a state of empathy can regulate attention processes. Previous research has shown that attention is guided according to the sensory-perceptual features of visual stimuli. In the present study, symbolic cues announcing painful electric stimulation for the partner elicited the brain signature of attentive processing, i.e., larger EPN and LPP components. This finding suggests empathy as a further avenue for the regulation of attention processes. There are noteworthy differences between these two avenues to guide selective attention processes in that empathy regulates attention in a fast and flexible way which is tailored to the given social context. More generally, understanding and feeling with somebody can temporarily highlight certain stimuli, possibly setting the stage for joint attention, cooperation, or helping behaviors. It will be interesting for future studies to examine whether and how this state of understanding and feeling with others vary with the social status of and attachment with the partner.

## Author Contributions

All authors contributed to the study concept. LW performed the data analysis and drafted the manuscript. HTS and TF provided critical revisions. Data collection was performed by GB, UK and AK. All authors approved the final version of the manuscript for submission.

## Conflict of Interest Statement

The authors declare that the research was conducted in the absence of any commercial or financial relationships that could be construed as a potential conflict of interest.

## Footnotes

^1^Exploratory analyses determined the relationship between the EPN and LPP difference scores (Pain—Nopain) for the partner condition in the anticipation phase and self-report measures of empathy. However, none of the correlations between differences in ERP amplitudes and empathy scores of the SPF, i.e., the sum score and the scores of the four subscales (i.e., Perspective taking, Fantasy, Empathetic concern and Personal distress), reached statistical significance (−0.12 ≤ *r*s ≤ 0.18, *p*s ≥ 0.32).

^2^Even though there was no indication for any modulation in the Partner-condition, exploratory *t*-tests were conducted to statistically corroborate this observation. Specifically, ERPs to the flash indicating the intensity of the stimulation for the partner did not differ for the N150–P260 (*t*_(31)_ = 1.64, *p* = 0.11), nor the P3 (*t*_(31)_ = 0.29, *p* = 0.77). Similarly, there were no significant effects of intensity of pain stimulation on the EPN (*t*_(31)_ = 0.53, *p* = 0.60) and LPP (*t*_(31)_ = 1.47, *p* = 0.15) in the Partner-condition.
